# Multimodal subspace independent vector analysis captures latent subspace structures in large multimodal neuroimaging studies

**DOI:** 10.1101/2023.09.17.558092

**Published:** 2023-09-17

**Authors:** Xinhui Li, Tulay Adali, Rogers Silva, Vince Calhoun

**Affiliations:** a Department of Electrical and Computer Engineering, Georgia Institute of Technology, Atlanta, GA, USA; b Tri-institutional Center for Translational Research in Neuroimaging and Data Science: Georgia State University, Georgia Institute of Technology, Emory University, Atlanta, GA, USA; c Department of Computer Science and Electrical Engineering, University of Maryland Baltimore County, Baltimore, MD, USA

**Keywords:** multimodal fusion, MSIVA, MISA, IVA

## Abstract

We present Multimodal Subspace Independent Vector Analysis (MSIVA), a methodology to capture both joint and unique vector sources across multiple data modalities by defining linked and modality-specific subspaces. In particular, MSIVA enables the estimation of independent subspaces of various sizes within modalities and their one-to-one linkage to corresponding subspaces across modalities. We compare MSIVA to a fully unimodal initialization baseline and a fully multimodal initialization baseline, and evaluate all three approaches with five distinct subspace structures on synthetic and neuroimaging datasets. We first demonstrate that MSIVA and the unimodal baseline can identify the correct ground-truth subspace structures from the incorrect ones in multiple synthetic datasets, while the multimodal baseline fails at detecting high-dimensional subspace structures. We then show that MSIVA can better capture the latent subspace structure with the minimum loss value in two large multimodal neuroimaging datasets compared to the unimodal baseline. Our results from subsequent per-subspace canonical correlation analysis (CCA) and brain-phenotype modeling demonstrate that the sources from the optimal subspace structure are strongly associated with phenotype measures, including age, sex and schizophrenia-related effects. Our proposed methodology MSIVA can be applied to capture linked and unique biomarkers from multimodal neuroimaging data.

## 1. Introduction

Multiple neuroimaging techniques such as magnetic resonance imaging (MRI) have been developed to uncover the structural and functional properties of the brain. However, each neuroimaging modality has its own strengths and weaknesses, and only captures limited information of the brain. For example, structural MRI (sMRI) can show high-resolution anatomical structure of the brain but cannot capture temporal dynamics, while functional MRI (fMRI) can measure blood-oxygenation-level-dependent (BOLD) signals across time at the cost of lower spatial resolution. Therefore, it is important to take advantage of multimodal neuroimaging to effectively capture interpretable and multifaceted information about the brain.

To jointly analyze multiple datasets or data modalities, a unified framework Multidataset Independent Subspace Analysis (MISA) [1] has been developed, encompassing multiple blind source separation methods, such as independent component analysis (ICA) [2], independent vector analysis (IVA) [3], and independent subspace analysis (ISA) [4]. MISA can be applied to identify latent sources from multiple neuroimaging modalities including sMRI and fMRI [1]. More recently, a multimodal IVA (MMIVA) fusion method built upon MISA has been proposed to identify linked biomarkers related to age, sex and psychosis in two large multimodal neuroimaging datasets [5]. MMIVA assumes that sources are independent within each modality, i.e. the subspace structure is an identity matrix. However, there may also exist linkage among sources in the same neuroimaging modality, potentially grouped by their anatomical or functional properties, which are not optimally captured by MMIVA.

Aiming to better detect linkage across sources and modalities, we present a novel methodology, *Multimodal Subspace Independent Vector Analysis (MSIVA)* [6], that captures linkage of vector sources by defining joint and unique subspaces. MSIVA is built upon MMIVA by defining a block diagonal matrix as the subspace instead of the identity matrix used in MMIVA. In addition, MSIVA is initialized with the weight matrices obtained by combining multimodal group principal component analysis (MGPCA) across modalities with separate ICAs for each modality. By design, MSIVA can simultaneously capture two types of latent sources: those linked across all modalities, as well as those unique to a specific modality.

We compare MSIVA with a fully unimodal initialization approach and a fully multimodal initialization approach. We demonstrate that MSIVA can successfully reconstruct both joint and unique sources, revealing the correct subspace structures in multiple synthetic datasets. We then apply MSIVA and the baseline approach on two large multimodal neuroimaging datasets, the UK Biobank dataset [7] and a mixed schizophrenia (SZ) patient dataset from several studies [8, 9, 10]. Our results indicate that MSIVA can better capture linked and modality-specific sources in the neuroimaging datasets with lower loss values compared to the baseline approach. Using canonical correlation analysis (CCA) [11], we conduct a follow-up assessment of each identified subspace separately and find projections within the optimal subspace structure yielding the post-CCA linked sources. We then perform an age regression task, a sex classification task, and an SZ classification task to evaluate the associations between these linked sources and phenotype measures. Results from brain-phenotype modeling suggest that the post-CCA sources are associated with age, sex and SZ-related effects.

## 2. Methods

### 2.1. Subspace Structure

Our interest lies on identifying groups of linked (i.e, *not* independent) sources within each modality, while assuming sources in different groups are statistically independent. These groups are referred to as *subspaces*. In addition, we aim to detect cross-modal linkage (i.e., statistical dependence) between subspaces. This requires solving a challenging combinatorial optimization problem. To simplify, we limit the search space of cross-modal linkages by assuming that links occur only between subspaces with the same size across modalities. Additionally, we assume all modality-specific subspaces to be 1D, i.e., a single source.

**Figure 1:**
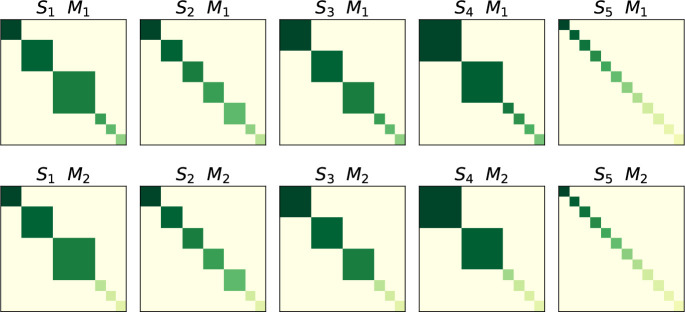
Five plausible candidate subspace structures $\left(S_{1}-S_{5}\right)$ for two modalities $\left(M_{1}-M_{2}\right)$. The top row shows the subspace structures for the first modality ($M_{1}$) while the bottom row shows the subspace structures for the second modality ($M_{2}$). Each panel depicts the idealized association between sources within each modality, across five different plausible scenarios. The block size represents the number of linked sources within modality (i.e., the subspace size). Same-color blocks are linked across modalities. The 1 × 1 blocks indicate modality-specific sources, except $S_{5}$, whose sources are one-to-one linked across two modalities.

Building on the MISA framework, we require a user-defined candidate subspace structure describing the expected linkage pattern. The goal of MSIVA is to determine which of the candidate subspace structures best fits the data. In particular, as shown in Figure 1, we propose five plausible subspace structures $\left(S_{1}-S_{5}\right)$ in two modalities $\left(M_{1}-M_{2}\right)$, all with 12 sources in each modality:

- $S_{1}$: One 2-dimensional (2D) linked subspace, one 3D linked subspace, one 4D linked subspace, and three 1D modality-specific subspaces.
- $S_{2}$: Five 2D linked subspaces and two 1D modality-specific subspaces.
- $S_{3}$: Three 3D linked subspaces and three 1D modality-specific subspaces.
- $S_{4}$: Two 4D linked subspaces and four 1D modality-specific subspaces.
- $S_{5}$: Twelve 1D linked subspaces (no modality-specific subspaces, as in MMIVA).

These choices are based on the functional imaging literature where two to four dimensions are commonly used to cluster functional networks [12, 13].

### 2.2. Multimodal Subspace Independent Vector Analysis

Given our constraint that linked cross-modal subspaces must have the same size, we refer to our proposed approach as *Multimodal Subspace Independent Vector Analysis (MSIVA)*. MSIVA is designed to capture both linked and modality-specific subspaces.

**Figure 2:**
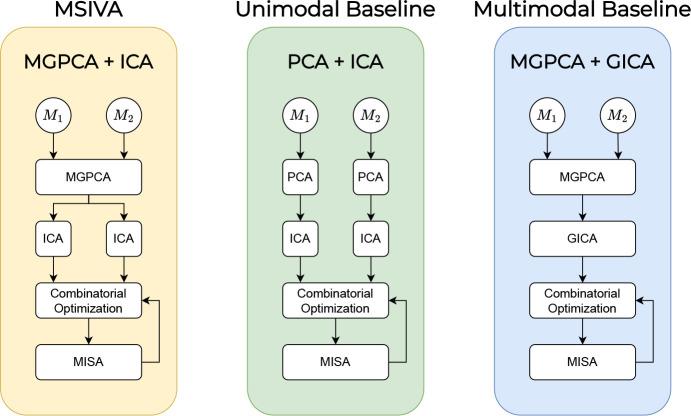
Overview of three proposed initialization methods. The initialization approaches from left to right are multimodal group PCA with separate ICAs per modality (MGPCA + ICA); separate PCAs followed by separate ICAs (PCA + ICA); multimodal group PCA with group ICA (MGPCA + GICA). The MGPCA + ICA initialization approach is denoted as MSIVA. After initialization, the combinatorial optimization and MISA are run for sufficient iterations until the loss value converges.

Three initialization workflows are considered for MSIVA, as shown in Figure 2. In all cases, given a candidate subspace structure, MSIVA consists of iterative combinatorial optimization (CO) of the source estimates (cross-modal subspace alignment) and numerical optimization of the MISA loss (see Equation 3). This process is repeated for each of five candidate subspace structures $\left(S_{1}-S_{5}\right)$ and followed by a best-fit determination based on the final loss values from all candidates. The following subsections explain each of these steps in detail.

#### 2.2.1. MSIVA Initialization

The MSIVA approach first utilizes multimodal group principal component analysis (MGPCA) to identify common principal components across two modalities and then applies separate ICA on the MGPCA-reduced data from each modality. The multimodal data matrices $X^{\left[m\right]}\in R^{V_{m}\times N}$ are reduced to C principal directions by MGPCA. Unlike principal component analysis (PCA) that identifies orthogonal directions of maximal variation for each modality separately, MGPCA identifies directions of maximal *common* variation across all modalities. Eigenvectors are computed based on the average of the *scaled* covariance matrices:

(1) $${\text{Σ}}_{\text{avg}\;}=\frac{1}{M}\sum_{m=1}^{M}N\frac{{\text{Σ}}^{\left[m\right]}}{\text{trace}\left({\text{Σ}}^{\left[m\right]}\right)}=\frac{1}{M}\sum_{m=1}^{M}N\frac{{X^{\left[m\right]}}^{⊤}X^{\left[m\right]}}{{\left\|X^{\left[m\right]}\right\|}_{\text{Fr}}^{2}},$$

where $\Sigma^{\left[m\right]}=\frac{X^{[m{]}^{⊤}}X^{\left[m\right]}}{V_{m}-1}\approx E[X^{[m{]}^{⊤}}X^{\left[m\right]}]$, E[⋅] is the expectation operator, and $∥\cdot {∥}_{Fr}$ indicates the Frobenius norm. The scaling factor $\frac{trace(\Sigma^{\left[m\right]})}{N}$ is the ratio of the variance in the modality to the number of samples. We define the whitening matrices ${whtM}^{\left[m\right]}$ as follows:

(2) $${whtM}^{\left[m\right]}=\sqrt{N-1}\Lambda^{-\frac{1}{2}}U^{[m{]}^{⊤}}k_{m},$$

where Λ and H are the top C eigenvalues (with the largest absolute values) and eigenvectors of $\Sigma_{\text{avg}}$, respectively, $U^{\left[m\right]}=\left(k_{m}X^{\left[m\right]}\right)H\Lambda^{-\frac{1}{2}}$, and $k_{m}=\sqrt{\frac{N}{M{∥X^{\left[m\right]}∥}_{Fr}^{2}}}=\sqrt{\frac{N\left(V_{m}-1\right)}{Mtrace(\Sigma^{\left[m\right]})}}$.

Next, the MGPCA-reduced data from each modality $X_{r}^{\left[m\right]}$ undergoes a separate ICA estimation using the Infomax objective [14] to obtain C independent sources ${\overset{ˆ}{s}}_{I}^{\left[m\right]}(n)=W_{I}^{\left[m\right]}X_{r}^{\left[m\right]}(n)$ per modality. These estimates are then optimized by running MISA as a unimodal ICA model initialized with $W_{F,0}^{\left[m\right]}=I$, leading to the final ICA source estimates ${\overset{ˆ}{s}}_{F}^{\left[m\right]}(n)=W_{F}^{\left[m\right]}x_{r}^{\left[m\right]}(n)$. Finally, multimodal MISA is initialized by the combined MGPCA+ICA estimates $W_{0}^{\left[m\right]}=W_{F}^{\left[m\right]}{whtM}^{\left[m\right]}$ from both modalities. Following, MSIVA is compared with a fully unimodal initialization approach and a fully multimodal initialization approach.

#### 2.2.2. Unimodal Initialization

The unimodal initialization approach simply applies PCA and ICA on each modality separately. We first project the data matrix from each modality $X^{\left[m\right]}$ into a reduced data matrix $X_{r}^{\left[m\right]}$ with C principal components. Next, we run ICA on each reduced data matrix $X^{\left[m\right]}$ to obtain C independent sources.

#### 2.2.3. Multimodal Initialization

The multimodal initialization approach sequentially applies MGPCA and group ICA (GICA) across all modalities. GICA performs ICA on the MGPCA reduced data from both modalities combined, i.e., $X_{r}=\sum_{m=1}^{M}\;X_{r}^{\left[m\right]}$.

#### 2.2.4. Alternating Combinatorial and Numerical Optimizations

All three approaches utilize MISA’s greedy combinatorial optimization (CO) and objective to recover sources. Greedy CO and MISA optimization are run iteratively for 10 and 20 iterations on synthetic and neuroimaging data, respectively, until the loss value converges. MISA uses the relative gradient and the L-BFGS [15] in a barrier-type optimization (fmincon from MATLAB’s Optimization Toolbox). Finally, we identify the optimal subspace structure based on the lowest loss value obtained. The loss function ℒ[1] [1] is defined as the Kullback-Leibler (KL) divergence between the joint Kotz distribution [16] of *all* sources p(s) and the product of the joint Kotz distribution of sources at each of K subspaces $q(s)=\prod_{k=1}^{K}\;p\left(s_{k}\right)$. Thus, subspaces are expected to be statistically independent of one another and may include multimodal sources. We want to minimize the loss ℒ by solving the following optimization problem:

(3) $$\begin{array}{l} \text{min}ℒ=\text{min}E\left[\text{ln}\frac{p\left(s\right)}{q\left(s\right)}\right]\\ \;=\text{min}E\left[\text{ln}p\left(s\right)\right]-\sum_{k=1}^{K}E\left[\text{ln}\;p\left(s_{k}\right)\right]\\ \;=\underset{\begin{array}{c} W,P_{k},k=1,\ldots ,K \end{array}}{\text{min}}E\left[\text{ln}\;p\left(Wx\right)\right]-\sum_{k=1}^{K}E\left[\text{ln}\;p\left(P_{k}Wx\right)\right], \end{array}$$

where W is the unmixing matrix such that s(n)=Wx(n) and $P_{k}$ is the *k*-th subspace assignment matrix defined by the subspace structure S.

The analysis code is publicly available at https://github.com/trendscenter/MSIVA.git.

### 2.3. Datasets

#### 2.3.1. Synthetic Data

For each subspace structure, we generate a synthetic dataset $X^{\left[m\right]}\in R^{V\times N}$, where m is the modality index (m∈{1,2}), V is the input feature dimensionality (V=20000) and N is the number of samples (N=3000). V and N are chosen to approximate the number of features and samples in the UK Biobank (UKB) neuroimaging dataset [7]. $X^{\left[m\right]}$ is a linear mixture of 12 sources spanning the defined subspaces. Each subspace is independently sampled from a multivariate Laplace distribution (the marginal distributions correspond to different sources). Sources in the same subspace, but assigned to different modalities, are dependent with a correlation coefficient ranging from 0.65 to 0.85. Sources in the 1D subspaces from $S_{1}$ to $S_{4}$ are independent from all others, i.e., correlation coefficient is 0.

#### 2.3.2. Neuroimaging Data

We utilize two large multimodal neuroimaging datasets including two image modalities: T1-weighted sMRI and fMRI. The first dataset is from UK Biobank [7]. 2907 subjects (mean age ± std: 62.09 ± 7.32 years; 1452 males, 1455 females) are used after excluding subjects missing phenotype measures. The second dataset includes 999 schizophrenia patients and controls (mean age ± std: 38.61 ± 13.13 years; 625 males, 374 females; 538 controls, 337 SZ patients, 124 patients with other mental disorders) combined from several studies (FBIRN [8], COBRE [9], BSNIP [10], MPRC). After subject selection, we preprocess these two imaging modalities to obtain the gray matter (GM) and amplitude of low frequency fluctuations (ALFF) feature maps, respectively. Data preprocessing details can be found in [5].

### 2.4. Experiments

For each of five subspace structures $\left(S_{1}-S_{5}\right)$, we first generate a synthetic dataset from the ground-truth subspace structure. Then we perform the experiments on all combinations of three initialization approaches and five subspace structures. Finally we measure the normalized multidataset Moreau-Amari intersymbol interference (ISI) [1, 17, 18], a metric to evaluate the residual differences between the recovered sources and the ground-truth sources, as well as the loss values. The synthetic data experiments aim to verify whether the proposed approaches including MSIVA can identify and distinguish the correct subspace structure used for data generation from the incorrect ones.

We then perform the same experiments on two multimodal neuroimaging datasets using these five candidate subspace structures, and identify the optimal structure yielding the lowest final MISA loss value. Separate follow-up CCA of each subspace recovered projections with maximum cross-modal correlation.

### 2.5. Phenotype Prediction

To further evaluate that association between the post-CCA sources and phenotype measures, we perform an age prediction task and a sex classification task for the UKB dataset, as well as an age prediction task and a binary SZ classification task (controls vs SZ patients) for the patient dataset. Specifically, we train a ridge regression model to predict the age, and a linear support vector machine to classify sex groups or SZ patients. For the UKB dataset, 2907 subjects are divided into a training set of 2000 subjects and a hold-out test set of 907 subjects. For the patient dataset, 999 subjects are divided into a training set of 699 subjects and a hold-out test set of 300 subjects in the age prediction task; 875 controls and SZ patients are grouped into a training set of 612 subjects and a test set of 263 subjects in the SZ diagnosis classification task. We perform 10-fold cross-validation to choose the best hyperparameters (regularization parameter range: [0.1*,* 1]) on the training set, then train the model using all training subjects and evaluate it on the hold-out test set. The age regression performance is measured by mean absolute error (MAE) and the classification performance is measured by accuracy.

## 3. Results

### 3.1. Synthetic Data Results

**Figure 3:**
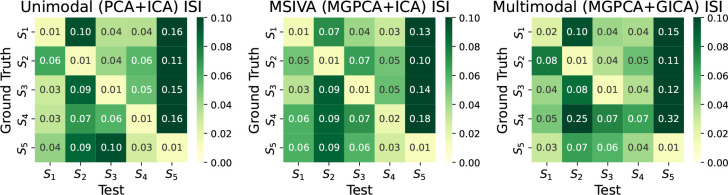
Synthetic data results: ISI (the lower the better). The unimodal initialization (PCA+ICA) and the MSIVA initialization (MGPCA+ICA) lead to the lowest ISI values (≤ 0.02) along the main diagonal, indicating that these two approaches can identify the correct ground-truth subspace structures from the incorrect ones. However, the multimodal initialization (MGPCA+GICA) fails at detecting the subspace structure $S_{4}$ with a high ISI value (0.07) in the main diagonal. Thus, MSIVA and the unimodal baseline are considered better than the multimodal baseline.

As shown in Figure 3, the unimodal initialization (PCA+ICA) and the MSIVA initialization (MGPCA+ICA) lead to the lowest ISI values (≤ 0.02) along the main diagonal, demonstrating that both approaches can correctly distinguish the ground-truth subspace structures from the incorrect ones. According to Table 1, the loss values are almost consistent with the ISI results, except that MSIVA misidentifies $S_{4}$. We also note that the loss value from MSIVA is slightly smaller than that from the unimodal initialization for each correct subspace structure, suggesting that MSIVA can fit the data slightly better than the unimodal initialization. However, the multimodal initialization with MGPCA and GICA fails at correctly detecting the ground-truth subspace structure with high-dimensional 4D subspace(s), i.e. $S_{1}$ and $S_{4}$. The multimodal initialization is considered worse due to the high ISI value (0.07) in the main diagonal and, thus, is excluded from neuroimaging data analysis according to the simulation results.

**Figure 4:**
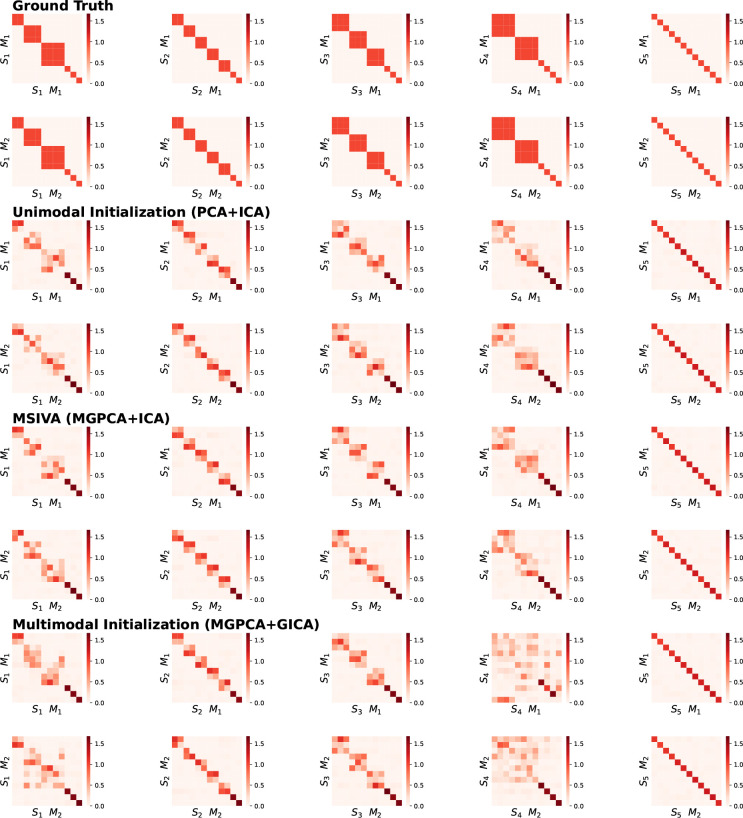
Synthetic data results: Interference matrices corresponding to diagonal ISI values in Figure 3. Correct subspace structures are identified and aligned across two modalities, following the ground-truth simulation design, except that the multimodal initialization (MGPCA+GICA) fails at reconstructing $S_{1}$ and $S_{4}$.

The recovered subspace structures from MSIVA and the unimodal initialization align with the proposed ground-truth ones (Figure 4). Block permutation of the subspaces is allowed, as long as alignment between modalities is retained (showing sorted result for ease of interpretation). Again, the multimodal initialization (MGPCA+GICA) fails at recovering the ground-truth subspace structures $S_{1}$ and $S_{4}$, suggesting that cross-modal alignment in high-dimensional subspaces may increase the difficulty of the optimization problem.

### 3.2. Neuroimaging Data Results

**Figure 5:**
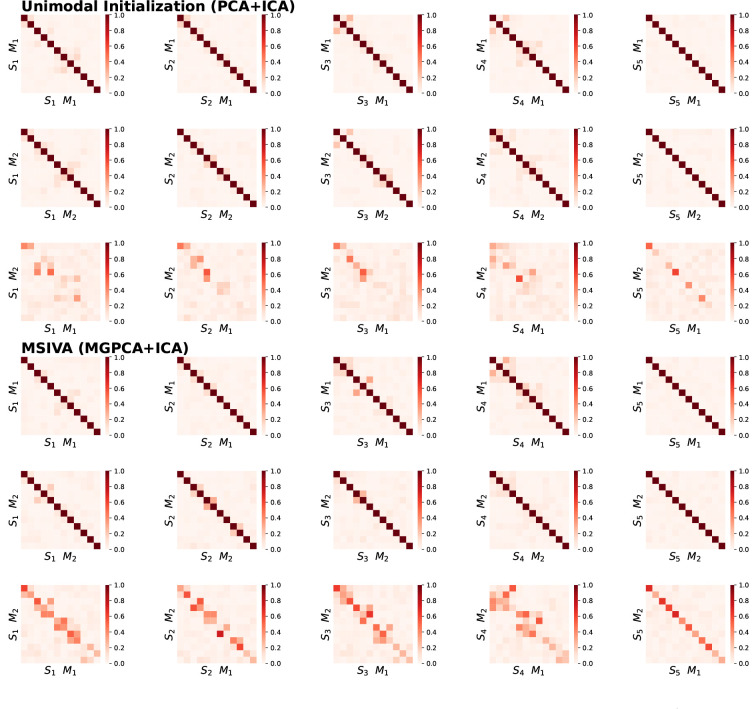
Neuroimaging data results: Within-modal and cross-modal correlations of the recovered sources for the UKB dataset. Within-modal self-correlation patterns align with the predefined subspace structures (rows I-II and IV-V). MSIVA recovers stronger cross-modal correlations than the unimodal baseline for all predefined subspace structures (row VI vs row III).

In the UKB neuroimaging dataset, we observe that within-modal self-correlation patterns align with the predefined subspace structures (Figure 5, rows I-II and IV-V). We note that MSIVA recovers stronger cross-modal correlation than the unimodal baseline for all predefined subspace structures (Figure 5, rows III and VI). MSIVA $S_{2}$ yields the lowest final MISA loss value 46.77 across all cases (Table 2), suggesting that the subspace structure $S_{2}$ best fits the latent structure of this dataset.

**Figure 6:**
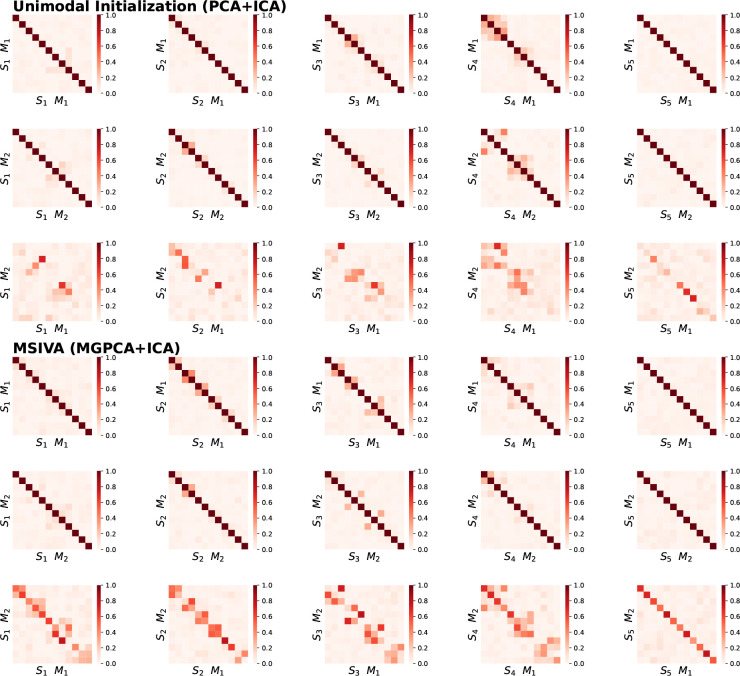
Neuroimaging data results: Within-modal and cross-modal correlations of the recovered sources for the patient dataset. Within-modal self-correlation patterns align with the predefined subspace structures (rows I-II and IV-V). MSIVA recovers stronger cross-modal correlations than the unimodal baseline for all predefined subspace structures (row VI vs row III).

Similarly, in the patient dataset, MSIVA shows stronger cross-modal correlation for all subspace structures (Figure 6, rows III and VI). Same as the UKB dataset, MSIVA $S_{2}$ yields the lowest final loss value 45.67 across all cases (Table 2).

**Figure 7:**
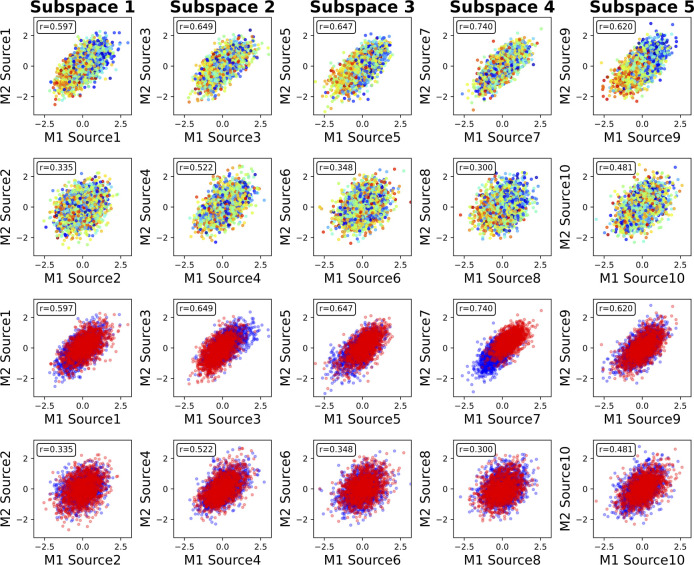
MSIVA $S_{2}$ linked UKB dataset sources, color coded by age and sex. Rows I and II show aging effect (warm color: older group; cold color: younger group). Rows III and IV show sex effect (blue: male; red: female). $M_{1}$: sMRI GM, $M_{2}$: fMRI ALFF. In particular, subspaces 1, 3, 4 and 5 are associated with aging (especially cross-modal source 9 in subspace 5), while subspaces 2 and 4 show sex effect (especially cross-modal source 7 in subspace 4).

**Figure 8:**
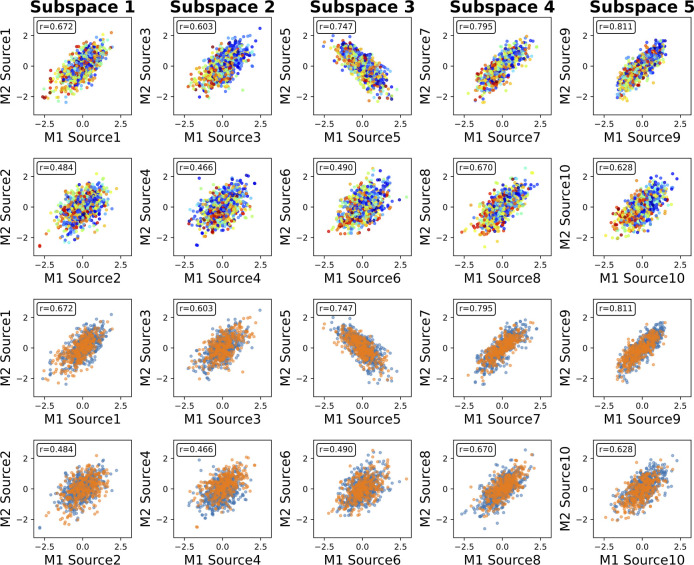
MSIVA $S_{2}$ linked patient dataset sources, color coded by age and diagnosis labels. Rows I and II show aging effect (warm color: older group; cold color: younger group). Rows III and IV show psychosis effect (light blue: controls; orange: schizophrenia patients). $M_{1}$: sMRI GM, $M_{2}$: fMRI ALFF. In particular, subspaces 2 and 5 are associated with aging (especially cross-modal source 9 in subspace 5) and SZ-related effects (especially cross-modal source 4 in subspace 2).

We then identify the associations between the phenotype measures and the sources captured by MSIVA $S_{2}$. In the UKB dataset, visual inspection of individual variability from the cross-modal CCA projections in each linked subspace (Figure 7) suggests that subspaces 1, 3, 4 and 5 are associated with aging (especially cross-modal source 9 in subspace 5), while subspaces 2 and 4 show sex effect (especially cross-modal source 7 in subspace 4). The age regression and sex classification results also confirm this finding (Table 3). Specifically, the age prediction mean absolute error (MAE) in subspace 5 is the lowest (5.400 years), and the sex classification accuracy is the highest in subspace 4 (0.812). In the patient dataset, we observe aging effect in source 3 from subspace 2 and source 9 from subspace 5, and psychosis effect in source 4 from subspace 2 and source 10 from subspace 5 according to cross-modal CCA projections in each linked subspace (Figure 8), verified by the age regression and diagnosis classification result (Table 3). No significant sex effect is found in the patient dataset.

**Figure 9:**
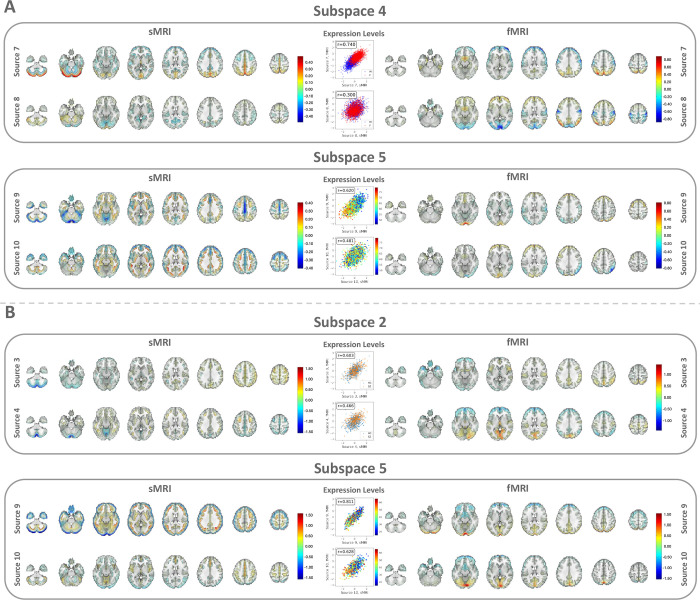
Spatial maps of MSIVA $S_{2}$ CCA projections related to phenotype measures. (A) UKB dataset spatial maps related to sex (source 7, subspace 4) and age (source 9, subspace 5). (B) Patient dataset spatial maps related to SZ (source 4, subspace 2) and age (source 10, subspace 5).

The dual-coded spatial maps [19] from sources in both modalities are presented in Figure 9, where the voxel intensity controls both the color and opacity. In the UKB dataset, source 7 from subspace 4 shows the strongest sex effect, while the source 9 from subspace 5 shows the strongest aging effect. The sex effect can be found in the occipital lobe (sMRI, fMRI), the frontal lobe (sMRI, fMRI) and the cerebellar region (sMRI). The age effect is shown in the cerebellar region (sMRI), the occipital lobe (fMRI), as well as the sensorimotor and visuomotor areas (sMRI, fMRI). In the patient dataset, the SZ effect can be found in the cerebellar area (sMRI), the temporal lobe (sMRI), the frontal lobe (sMRI, fMRI) and the occipital lobe (sMRI, fMRI). The age effect can be seen in the occipital lobe (sMRI, fMRI) and the frontal lobe (fMRI).

## 4. Discussion

We present a novel methodology, Multimodal Subspace Independent Vector Analysis (MSIVA), to capture linked cross-modal sources as well as modality-specific sources. We first show that both MSIVA and the unimodal baseline can correctly identify the ground-truth subspace structures from the incorrect ones, and verify that the correct subspace structures result in the lowest loss values from multiple synthetic data experiments. We next apply both approaches on two large multimodal neuroimaging datasets and demonstrate that MSIVA can better reveal the latent subspace structure across two imaging modalities with lower loss values. Among all cases, MSIVA $S_{2}$ outputs the lowest loss value and, thus, is considered as the best fit to the latent structure in both neuroimaging datasets. The CCA projections within each shared subspace are strongly associated with age, sex and SZ-related effects, as verified through the phenotype prediction tasks. The age, sex and SZ-related spatial maps align with previous findings [5].

Furthermore, MSIVA can be viewed as an extension of MMIVA except that their initialization methods (MSIVA: MGPCA+ICA initialization; MMIVA: MGPCA+GICA initialization) and subspace structures (MSIVA: flexible subspace structures; MMIVA: $S_{5}$) are different. To further investigate the estimated sources from MSIVA and MMIVA, we compare MSIVA with the subspace structure $S_{2}$ and MMIVA by using MSIVA $S_{2}$ sources to predict MMIVA sources, as well as using matched MMIVA sources to predict MSIVA $S_{2}$ sources. We find that every two MSIVA $S_{2}$ sources from each subspace can predict more than two MMIVA sources, while every two matched MMIVA sources can also predict more than two MSIVA $S_{2}$ sources (see Appendix A). Thus, there is no one-to-one mapping between MSIVA $S_{2}$ sources and MMIVA sources. MSIVA and MMIVA estimate sources in different ways.

It is important to acknowledge limitations of our current work. The first limitation is the subspace structure used in MSIVA. MSIVA selects the subspace structure that best fits the data from a predefined set, according to the loss value. However, it is not efficient to exhaustively evaluate potential subspace structures and corresponding performance metrics. Additionally, we make two assumptions on the subspace structure that the linked subspaces share the same size across modalities, and that the modality-specific subspaces are one-dimensional. However, it is possible that these assumptions may not represent the underlying data structure. Ideally, we want to learn the underlying subspace structure from the data in an unsupervised manner. The second limitation is the linear mixing assumption in MSIVA. MSIVA assumes that the data of each modality can be transformed to linearly mixed sources, but the true mixing process can be nonlinear. Also, individual differences, site effects and preprocessing-related effects may introduce nonlinear artifacts, which are not taken into account in MSIVA. Future work will include applying data-driven subspace structures such as NeuroMark template [20], and developing nonlinear unsupervised learning algorithms to automatically estimate the optimal subspace structure.

## 5. Conclusions

Our proposed approach MSIVA can capture linked and modality-specific sources on both synthetic and neuroimaging datasets and yield a lower loss value compared to the unimodal baseline. The sources in the linked subspaces are strongly associated with age, sex and psychosis according to brain-phenotype modeling. Our results support that MSIVA can be applied to capture linked and unique imaging biomarkers from large multimodal neuroimaging datasets.

## Figures and Tables

**Table 1:** Synthetic data results: Final MISA loss values (the lower the better). Each row represents a ground-truth (GT) subspace structure used to generate the data and each column represents a test subspace structure used to fit the model. The lowest loss value along the row is highlighted in bold, which determines the selected subspace. Approaches performing consistently well will contain bold loss values *only* along the diagonal. The unimodal initialization approach correctly identifies all five subspace structures. MSIVA correctly identifies all cases except for $S_{4}$. The multimodal initialization approach fails at detecting $S_{1}$ and $S_{4}$.

Unimodal Initialization (PCA+ICA)	$S_{1}^{\text{Test}}$	$S_{2}^{\text{Test}}$	$S_{3}^{\text{Test}}$	$S_{4}^{\text{Test}}$	$S_{5}^{\text{Test}}$
$S_{1}^{\text{GT}}$	**42.69**	42.88	42.76	42.99	43.23
$S_{2}^{\text{GT}}$	42.65	**42.30**	42.85	42.87	42.92
$S_{3}^{\text{GT}}$	42.72	42.86	**42.64**	43.10	43.26
$S_{4}^{\text{GT}}$	43.09	43.24	43.17	**42.98**	43.51
$S_{5}^{\text{GT}}$	43.40	43.01	43.50	43.77	**42.02**
MSIVA (MGPCA+ICA)	$S_{1}^{\text{Test}}$	$S_{2}^{\text{Test}}$	$S_{3}^{\text{Test}}$	$S_{4}^{\text{Test}}$	$S_{5}^{\text{Test}}$
$S_{1}^{\text{GT}}$	**42.68**	42.86	42.75	43.04	43.11
$S_{2}^{\text{GT}}$	42.66	**42.23**	42.63	42.76	42.75
$S_{3}^{\text{GT}}$	42.69	42.86	**42.62**	43.04	43.13
$S_{4}^{\text{GT}}$	42.69	42.40	41.12	39.94	**33.61**
$S_{5}^{\text{GT}}$	43.41	42.97	43.39	43.98	**42.01**
Multimodal Initialization (MGPCA+GICA)	$S_{1}^{\text{Test}}$	$S_{2}^{\text{Test}}$	$S_{3}^{\text{Test}}$	$S_{4}^{\text{Test}}$	$S_{5}^{\text{Test}}$
$S_{1}^{\text{GT}}$	**23.82**	23.95	**23.82**	24.03	24.27
$S_{2}^{\text{GT}}$	27.77	**27.44**	27.80	28.16	28.18
$S_{3}^{\text{GT}}$	23.93	24.03	**23.78**	24.04	24.23
$S_{4}^{\text{GT}}$	**17.26**	18.66	17.29	17.56	19.73
$S_{5}^{\text{GT}}$	36.76	36.36	36.75	37.26	**35.26**

**Table 2:** Neuroimaging data results: Final MISA loss values (the lower the better). MSIVA with the subspace structure $S_{2}$ outputs the lowest loss values in both multimodal neuroimaging datasets, and thus is considered as the optimal approach to capture the latent subspace structure in neuroimaging data.

Subspace	$S_{1}$	$S_{2}$	$S_{3}$	$S_{4}$	$S_{5}$
UK Biobank Dataset					
Unimodal Initialization (PCA+ICA)	47.74	47.81	47.77	47.78	48.00
MSIVA (MGPCA+ICA)	46.79	**46.77**	46.80	46.89	46.92
Patient Dataset					
Unimodal Initialization (PCA+ICA)	47.36	47.35	47.34	47.40	47.53
MSIVA (MGPCA+ICA)	45.77	**45.67**	45.79	45.92	45.70

**Table 3:** Phenotype prediction performance using MSIVA $S_{2}$ linked subspaces. For the UKB dataset, sources from subspaces 5 and 4 yield the best age regression and sex classification performance, respectively. For the patient dataset, sources from subspaces 5 and 2 yield the best age regression and SZ classification performance, respectively. These linked sources from MSIVA $S_{2}$ are strongly associated with age, sex, and SZ-related effects.

Subspace	1	2	3	4	5
UK Biobank dataset					
Age MAE	5.757	6.270	5.936	5.888	**5.400**
Sex accuracy	0.594	0.610	0.576	**0.812**	0.530
Patient dataset					
Age MAE	10.694	9.632	10.616	10.738	**9.461**
Diagnosis accuracy (SZ vs HC)	0.597	**0.677**	0.608	0.597	0.639

**Figure A.10:**
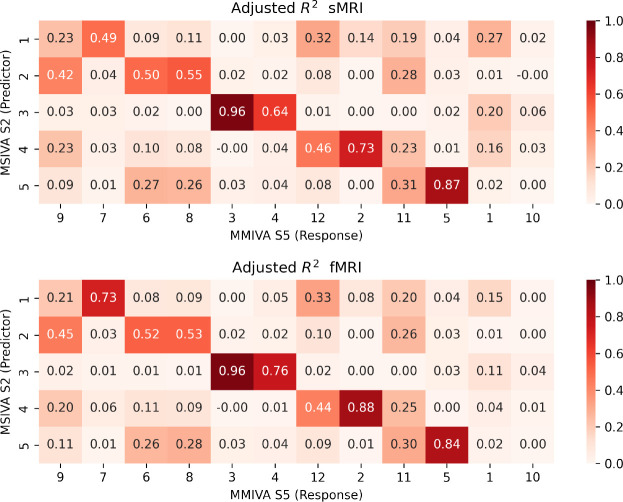
Adjusted $R^{2}$ using MSIVA $S_{2}$ sources to predict MMIVA sources for the UKB dataset.

**Figure A.11:**
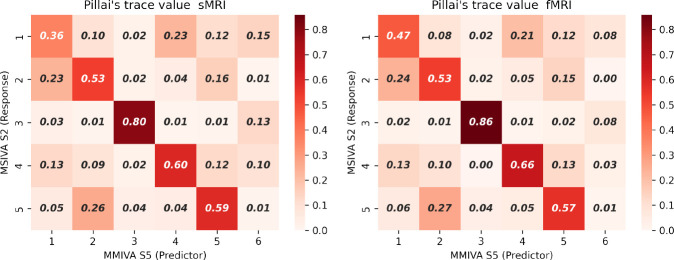
Pillai’s trace value using MMIVA sources to predict MSIVA $S_{2}$ sources for the UKB dataset.

**Figure A.12:**
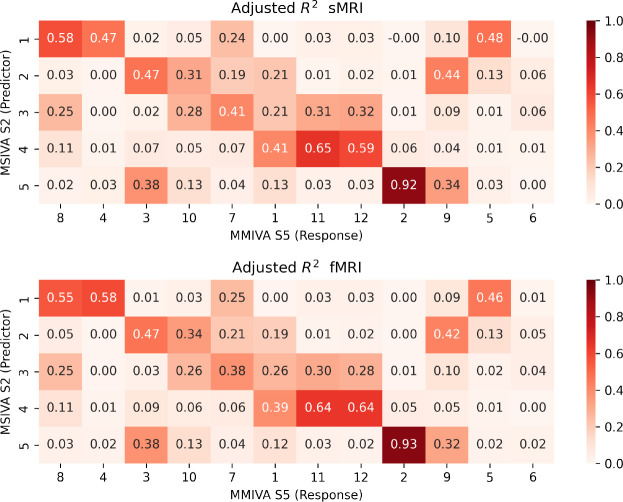
Adjusted $R^{2}$ using MSIVA $S_{2}$ sources to predict MMIVA sources for the patient dataset.

**Figure A.13:**
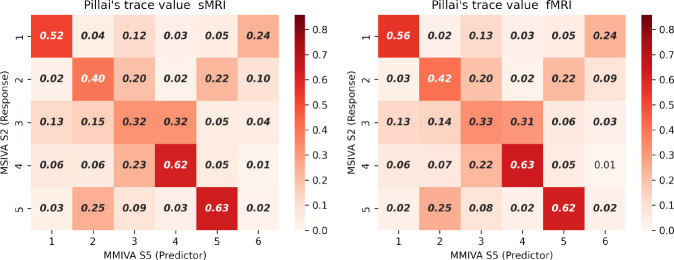
Pillai’s trace value using MMIVA sources to predict MSIVA $S_{2}$ sources for the patient dataset.

## Appendix A. Comparison between MSIVA S2 and MMIVA

MSIVA can be viewed as an extension of MMIVA with two main differences. First, MSIVA uses a *block* diagonal subspace structure while MMIVA uses an identity matrix as the subspace structure ($S_{5}$). Second, MSIVA uses MGPCA and separate ICAs initialization while MMIVA uses MGPCA and group ICA initialization. To further investigate similarities and differences of the recovered sources from MSIVA and MMIVA, we compare MSIVA with the subspace structure $S_{2}$ and MMIVA through following experiments: 1) using MSIVA $S_{2}$ sources to predict MMIVA sources; 2) using matched MMIVA sources to predict MSIVA $S_{2}$ sources.

Figures A.10 and A.12 show the adjusted $R^{2}$ when using every two MSIVA $S_{2}$ sources from each subspace to predict each of 12 MMIVA sources for the UKB dataset and the patient dataset, respectively. We reorder MMIVA sources to identify the most likely correspondence between MSIVA $S_{2}$ sources and MMIVA sources. We note that there exists correspondence between MSIVA $S_{2}$ sources and MMIVA sources. For example, for UKB sMRI data, MSIVA $S_{2}$ subspace 3 sources match MMIVA source 3 $\left(R_{adj}^{2}=0.96\right)$, MSIVA $S_{2}$ subspace 5 sources match MMIVA source 5 $\left(R_{adj}^{2}=0.87\right)$, and MSIVA $S_{2}$ subspace 4 sources match MMIVA source 2 $\left(R_{adj}^{2}=0.73\right)$. We also observe that there are more than two columns showing high $R_{adj}^{2}(>0.2)$ for each row, indicating that every two MSIVA $S_{2}$ sources from each subspace can predict more than two MMIVA sources.

We then perform a multivariate prediction by using every two matched MMIVA sources to predict every two MSIVA $S_{2}$ sources from each subspace. We show the Pillai’s trace divided by the number of modalities, which is 2, in Figures A.11 and A.13. Note that the maximum diagonal Pillai’s trace value is 2 for two modalities, and dividing the Pillai’s trace by 2 shows variance explained for each modality. We observe large off-diagonal values per column, indicating that every two matched MMIVA sources can predict more than two MSIVA $S_{2}$ sources.

Thus, we conclude that there is no one-to-one mapping between MSIVA $S_{2}$ sources and MMIVA sources. MSIVA and MMIVA estimate sources in different ways. It is not a fair comparison using paired MMIVA sources to predict MSIVA $S_{2}$ sources or vice versa for downstream analyses.

## References

[1] Silva R. F., Plis S. M., Adalı T., Pattichis M. S., Calhoun V. D., Multidataset independent subspace analysis with application to multimodal fusion, IEEE Transactions on Image Processing 30 (2020) 588–602. 33031036 10.1109/TIP.2020.3028452 PMC7877797

[2] Comon P., Independent component analysis, a new concept?, Signal processing 36 (3) (1994) 287–314.

[3] Kim T., Eltoft T., Lee T.-W., Independent vector analysis: An extension of ica to multivariate components, in: International conference on independent component analysis and signal separation, Springer, 2006, pp. 165–172.

[4] Cardoso J.-F., Multidimensional independent component analysis, in: Proceedings of the 1998 IEEE International Conference on Acoustics, Speech and Signal Processing, ICASSP’98 (Cat. No. 98CH36181), Vol. 4, IEEE, 1998, pp. 1941–1944.

[5] Silva R. F., Damaraju E., Li X., Kochunov P., Belger A., Ford J. M., Mathalon D. H., Mueller B. A., Potkin S. G., Preda A., , Direct linkage detection with multimodal iva fusion reveals markers of age, sex, cognition, and schizophrenia in large neuroimaging studies, bioRxiv (2021) 2021–12.

[6] Li X., Adali T., Silva R. F., Calhoun V. D., Multimodal subspace independent vector analysis better captures hidden relationships in multimodal neuroimaging data, in: 2023 IEEE 20th International Symposium on Biomedical Imaging (ISBI), IEEE, 2023, pp. 1–5.

[7] Miller K. L., Alfaro-Almagro F., Bangerter N. K., Thomas D. L., Yacoub E., Xu J., Bartsch A. J., Jbabdi S., Sotiropoulos S. N., Andersson J. L., , Multimodal population brain imaging in the uk biobank prospective epidemiological study, Nature neuroscience 19 (11) (2016) 1523–1536. 27643430 10.1038/nn.4393 PMC5086094

[8] Keator D. B., van Erp T. G., Turner J. A., Glover G. H., Mueller B. A., Liu T. T., Voyvodic J. T., Rasmussen J., Calhoun V. D., Lee H. J., , The function biomedical informatics research network data repository, Neuroimage 124 (2016) 1074–1079. 26364863 10.1016/j.neuroimage.2015.09.003 PMC4651841

[9] Aine C., Bockholt H. J., Bustillo J. R., Cañive J. M., Caprihan A., Gasparovic C., Hanlon F. M., Houck J. M., Jung R. E., Lauriello J., , Multimodal neuroimaging in schizophrenia: description and dissemination, Neuroinformatics 15 (4) (2017) 343–364. 28812221 10.1007/s12021-017-9338-9 PMC5671541

[10] Tamminga C. A., Pearlson G., Keshavan M., Sweeney J., Clementz B., Thaker G., Bipolar and schizophrenia network for intermediate phenotypes: outcomes across the psychosis continuum, Schizophrenia bulletin 40 (Suppl_2) (2014) S131–S137. 24562492 10.1093/schbul/sbt179 PMC3934403

[11] Hotelling H., Relations between two sets of variates, in: Breakthroughs in statistics, Springer, 1992, pp. 162–190.

[12] Ma S., Correa N. M., Li X.-L., Eichele T., Calhoun V. D., Adali T., Automatic identification of functional clusters in fmri data using spatial dependence, IEEE Transactions on Biomedical Engineering 58 (12) (2011) 3406–3417. 21900068 10.1109/TBME.2011.2167149 PMC3222740

[13] Ma S., Li X.-L., Correa N. M., Adali T., Calhoun V. D., Independent subspace analysis with prior information for fmri data, in: 2010 IEEE International Conference on Acoustics, Speech and Signal Processing, IEEE, 2010, pp. 1922–1925.

[14] Bell A. J., Sejnowski T. J., An information-maximization approach to blind separation and blind deconvolution, Neural computation 7 (6) (1995) 1129–1159. 7584893 10.1162/neco.1995.7.6.1129

[15] Liu D. C., Nocedal J., On the limited memory bfgs method for large scale optimization, Mathematical programming 45 (1–3) (1989) 503–528.

[16] Kotz S., Multivariate distributions at a cross road, in: A Modern Course on Statistical Distributions in Scientific Work, Springer, 1975, pp. 247–270.

[17] Macchi O., Moreau E., Self-adaptive source separation by direct or recursive networks, in: Proc ICDSP 1995, Cyprus, 1995, pp. 122–129.

[18] Amari S.-I., Cichocki A., Yang H. H., A New Learning Algorithm for Blind Signal Separation, Proc NIPS 1996 8 (1996) 757–763.

[19] Allen E. A., Erhardt E. B., Calhoun V. D., Data visualization in the neurosciences: overcoming the curse of dimensionality, Neuron 74 (4) (2012) 603–608. 22632718 10.1016/j.neuron.2012.05.001 PMC4427844

[20] Du Y., Fu Z., Sui J., Gao S., Xing Y., Lin D., Salman M., Abrol A., Rahaman M. A., Chen J., , Neuromark: An automated and adaptive ica based pipeline to identify reproducible fmri markers of brain disorders, NeuroImage: Clinical 28 (2020) 102375. 32961402 10.1016/j.nicl.2020.102375 PMC7509081

